# Decoding Poultry Welfare from Sound—A Machine Learning Framework for Non-Invasive Acoustic Monitoring

**DOI:** 10.3390/s25092912

**Published:** 2025-05-05

**Authors:** Venkatraman Manikandan, Suresh Neethirajan

**Affiliations:** 1 Faculty of Computer Science, Dalhousie University, Halifax, NS B3H 4R2, Canada; 2 Faculty of Agriculture, Dalhousie University, Halifax, NS B3H 4R2, Canada

**Keywords:** poultry vocalizations, acoustic monitoring, animal welfare, machine learning, precision farming, bioacoustics, mfcc features, stress detection, time-series models, interpretable AI

## Abstract

Acoustic monitoring presents a promising, non-invasive modality for assessing animal welfare in precision livestock farming. In poultry, vocalizations encode biologically relevant cues linked to health status, behavioral states, and environmental stress. This study proposes an integrated analytical framework that combines signal-level statistical analysis with machine learning and deep learning classifiers to interpret chicken vocalizations in a welfare assessment context. The framework was evaluated using three complementary datasets encompassing health-related vocalizations, behavioral call types, and stress-induced acoustic responses. The pipeline employs a multistage process comprising high-fidelity signal acquisition, feature extraction (e.g., mel-frequency cepstral coefficients, spectral contrast, zero-crossing rate), and classification using models including Random Forest, HistGradientBoosting, CatBoost, TabNet, and LSTM. Feature importance analysis and statistical tests (e.g., *t*-tests, correlation metrics) confirmed that specific MFCC bands and spectral descriptors were significantly associated with welfare indicators. LSTM-based temporal modeling revealed distinct acoustic trajectories under visual and auditory stress, supporting the presence of habituation and stressor-specific vocal adaptations over time. Model performance, validated through stratified cross-validation and multiple statistical metrics (e.g., F1-score, Matthews correlation coefficient), demonstrated high classification accuracy and generalizability. Importantly, the approach emphasizes model interpretability, facilitating alignment with known physiological and behavioral processes in poultry. The findings underscore the potential of acoustic sensing and interpretable AI as scalable, biologically grounded tools for real-time poultry welfare monitoring, contributing to the advancement of sustainable and ethical livestock production systems.

## 1. Introduction and Background

In recent years, sensor-based monitoring systems have been at the forefront of research in the field of animal welfare advancement, health diagnostics, and behavioral investigation. Among all possible sensing modalities, acoustic monitoring has become a very powerful tool due to its non-invasive means of capturing biologically meaningful signals that reflect the internal states of animals. Vocalizing, jaw movements, and respiratory sounds collected through microphones or acoustic sensors can provide a continuous and real-time view of an animal’s physiological and emotional states.

Bioacoustic analyses have proved their potential across different species. Using acoustic sensors for jaw movement detection, for example, has been shown to accurately identify grazing and ruminating behaviors of beef cattle, providing a very fine-scale assessment method for welfare analysis in open environments [1]. Identical to the use of facial detection methods in cattle—such as the Cow Face Detection Network (CFDN)—these create individualized monitoring by integrating visual sensors into deep learning, thus advancing precision livestock management [2].

Beyond cows, these systems for deep learning-based acoustic classification have also been employed to vocalizations from elephants, allowing researchers to interpret their communication patterns based on their behaviors through spectrogram-based CNN architectures [3]. An automated voice analysis in dogs also has the capacity to identify the vocal biomarkers of emotional arousal, stress, and breed-specific characteristics, which are useful for veterinary diagnostics as well as welfare assessment [4].

In poultry, vocalizations are tightly coupled with stress, disease, hunger, and social interactions, making chickens an ideal candidate species for acoustic sensing. Microphones can passively sample an entire flock rather than disrupting their natural behavior while visual equipment might only capture a few birds at a time. Previous studies have indicated that statistical features drawn from poultry vocalizations, such as mel-frequency cepstral coefficients (MFCCs), spectral entropy, and zero-crossing rate, can be incorporated into machine learning models in a meaningful way for classifying health conditions, behavioral states, and responses to environmental stimuli.

While promising, there is much existing work that focuses strictly on classification accuracy while giving no attention to the interpretability, statistical significance, and biological validity of the features used. Another area that demands attention is the generalization to real-world settings, which is limited by environmental noise, dataset variability, and a dearth of feature-wise analysis. Furthermore, comparative evaluation amongst different ML models, particularly with respect to ensemble and deep learning methods, has been sparse in terms of acoustic poultry datasets.

Our approach, unlike past methods, does not just treat the entire acoustic model as a black box classification task but also combines signal-level statistical tests with machine learning classifiers for health, behavior, and stressor-specific classification tasks. It focuses on diverse models, for example, CatBoost, MLPClassifier, Random Forest, and TabNet, across multiple datasets and conditions, providing insight into model-level strengths and weaknesses. Specifically, we demonstrate how specific acoustic parameters relate to stress, disease, and behavioral expression, thus increasing the scope of applicability and explanatory power of acoustic sensing in poultry welfare systems by integrating statistical modeling, machine learning, and domain knowledge. This work helps further integrate real-time deployments for non-invasive poultry welfare monitoring.

### 1.1. Poultry Vocalization Analysis and Health Monitoring

Poultry vocalizations represent a rich, non-invasive source for automatically monitoring welfare, health, and behavioral states. Recent developments in sensing technologies and deep learning have enabled the widespread collection and analysis of these acoustic signals. The statistical parameters from vocalizations—including features such as spectral centroid, bandwidth of energy, formant structure, and entropy—are constantly making their way into machine learning models for real-time classified detection tasks. 

Carvalho et al. [5] developed a CNN-based classification of broiler vocalizations into pleasure notes, distress calls, short peeps, and warbles, with an average accuracy of 91.1%. Adebayo et al. [6] used signal processing and machine learning to distinguish coughing and snoring in chickens. Zhao et al. [7] demonstrated how sound pressure levels affect vocal output in chicks. Srinivasagan et al. [8] used TinyML on edge devices, achieving 96.6% accuracy. Cuan et al. [9,10] proposed CNN-based models for detecting Newcastle disease and avian influenza from chicken vocalizations. Du et al. [11] linked vocal types with thermal stress using SVM. Hassan et al. [12] incorporated noise into training, achieving high accuracy with reduced model size.

Ginovart-Panisello et al. [13,14,15,16,17] conducted multiple studies analyzing CO_2_ levels, fasting stress, and vaccination effects on broiler chicken vocalizations. Li et al. [18] used spectrogram-based deep learning for chick sex detection. Schober et al. [19] explored vocal repertoires in ducks. Collins et al. [20] studied vocal changes in chicks under emotional stress. 

### 1.2. Cross-Species Applications and Bioacoustic Modeling 

Deep learning-based acoustic classification has also been employed across species. Jung et al. [21] achieved 90% accuracy in cattle vocalization classification using CNNs. Wang et al. [22] identified sheep behaviors acoustically. Ivanenko et al. [23] and Premoli et al. [24] classified ultrasonic mouse vocalizations with CNNs and wavelet spectrograms, revealing emotional and genetic traits. Bravo Sanchez et al. [25] built an end-to-end model for raw waveform-based bird call classification. Gavojdian et al. [26] developed BovineTalk for identifying cow stress calls, while Sattar [27] used MSVM and cochleagram features for cow behavior recognition. 

CASE, developed by Schneider et al. [28], is an unsupervised framework for comparing clustering algorithms in vocal data. Lavner and Pérez-Granados [29] highlighted the importance of generalization, interpretability, and hybrid architecture. Mutanu et al. [30] reviewed 124 studies and noted dominant trends in CNN and ensemble methods in bioacoustics. 

### 1.3. Advances in Birdsong Analysis, Transfer Learning, and Foundations

Bird-specific tools like BirdNET have become popular. Terasaka et al. [31] benchmarked BirdNET against other segmentation tools. Sethi et al. [32] used BirdNET to detect bird species across four countries. McGinn et al. [33] employed BirdNET embeddings to differentiate intraspecies calls. 

Ghani et al. [34] applied transfer learning and knowledge distillation on the xeno-canto dataset. Mørk et al. [35] used Data2Vec for noise-robust keyword spotting. Sasek et al. [36] built a DSSS model for separating bird vocalizations from site-specific noise. Brydinskyi et al. [37] showed wav2vec2 fine-tuning can improve ASR accuracy. Shirahata et al. [38] introduced a Whisper-based model for phonemic and prosodic annotation. 

Tosato et al. [39] demonstrated that AutoKeras outperforms handcrafted models for bird classification. Early foundational studies by Collias and Joos [40] spectrographed distress, pleasure, and alarm calls in chickens. More recently, the authors of [41] focused on unsupervised learning and label refinement in large bird call datasets. 

The rest of the article has the following structure. In Section 2, we describe in detail the datasets used in the analysis as well as explain the acoustic sensing and applied machine learning techniques, and how they were used in the study. In Section 3, the results of the experiments, such as classification results, feature relevance analysis, and biological interpretation over various datasets, are discussed. Section 4 critiques the shortcomings in the approach taken and offers suggestions for new explorative frameworks to be pursued in future work. The paper is concluded in Section 5, where the primary remarks and arguments made in the paper are presented along with an overview of the proposed method’s capabilities in real-time, non-invasive assessments of poultry welfare.

## 2. Materials and Methods

### 2.1. Dataset Descriptions

Dataset 1: Chicken Vocalization for Health Detection

The Zenodo dataset [https://zenodo.org/records/10433023 (accessed on 6 April 2025)] contains annotated audio clips belonging to broiler chickens recorded under controlled experimental conditions. The main aim of the dataset is to distinguish between healthy and unhealthy birds. In total, the dataset has 52 labeled audio samples classified into two classes (28 healthy and 24 unhealthy) each denoting general health and disease situations such as respiratory infection. Recording took place in low-noise laboratory environments, using directional microphones sampling at 44.1 kHz. The average duration of each clip is approximately 5–10 s. All files were converted to mono and standardized in sample rate prior to feature extraction. Spectral subtraction and silence trimming were applied to signals so that preprocessing improved signal clarity. 

Dataset 2: Chicken Language Dataset

The ChickenLanguageDataset [https://github.com/zebular13/ChickenLanguageDataset (accessed on 6 April 2025)] consists of a very huge collection of audio samples capturing chicken vocalizations in different behavioral and social contexts. This dataset contains labeled samples for vocal types like tidbitting, do_you_have_food, where_is_everyone, hungry, and eating. The recordings were taken using electret condenser microphones installed in a pen with little human interference, in natural ambient noise and lighting. The audio files were sampled at 48 kHz and last from 2 to 7s. This dataset was intended primarily for tasks involving behavioral classification. Noise gating and dynamic range normalizations were carried out before modeling to remove external noise, and the final class-wise distribution was checked for imbalance before under-represented classes were kept after synthetic balancing using stratified sampling during cross-validation. 

Dataset 3: Stress Detection in Poultry 

This dataset contains the vocal responses of chickens exposed to both visual stressors (the opening of umbrellas) and auditory stressors (dog barking). Longitudinal recordings were taken in different weeks to track adjustment or sensitization over time to that stress. The dataset contains more than 200 audio segments labeled according to the type of stressor and the week of recording. The placing of microphones was strategic enough to catch the vocalization responses while avoiding too much interference from equipment. During preprocessing, partial mitigation of background noise such as barn fans was achieved through high-pass filtering and spectral noise reduction. On average each clip lasts around 4–6 s. This dataset enabled time-series trend analysis of stress-induced vocal features.

All animal vocalization recordings used in this study complied with the respective institutional animal care and use guidelines. No experimental harm or behavioral disruption was introduced.

### 2.2. Acoustic Sensing Framework in Precision Poultry Monitoring

Sensors are key instruments in advancing precision livestock systems, making it possible to non-invasively inspect animal health and welfare in the real world. Advantageous to poultry production, acoustic sensing provides a medium for the utmost purpose, since birds’ vocalizations house rich, quantifiable cues about physiological state, emotional responses, or behavioral changes. In contrast to visual monitoring or tagging with wearables, microphones are inexpensive, scalable, and can continuously monitor the behavior of huge flocks without them knowing or causing stress.

**Figure 1:**
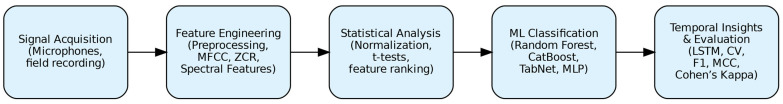
Acoustic sensing pipeline for poultry monitoring, including signal acquisition, preprocessing, feature extraction, statistical analysis, and machine learning-based classification of health, behavior, and stress states.

In this study, acoustic sensing formed the foundation of a data-driven pipeline (Figure 1) for evaluating poultry welfare. The audio signals were recorded in controlled environments using external microphones set for high-fidelity recording. These vocalizations, taking place under certain behaviors or physiological conditions (feeding, distress, health deterioration), are natural outputs of internal states and, therefore, passive signals that biologically give information about the animals’ states.

The pipeline for analyzing this acoustic data has four key stages:

Signal Acquisition: High-fidelity audio recordings were made at poultry farms or research environments using condenser microphones set at strategic locations for good pickup of vocal outputs.

Preprocessing: The raw signals were cleaned and segmented using different methods like spectral subtraction and windowed framing, so that background noise could be eliminated, enhancing the quality of features.

Feature Extraction: Acoustic features were computed from the recordings, including mel-frequency cepstral coefficients (MFCCs), spectral contrast, zero-crossing rate (ZCR), and a number of other temporal and spectral descriptors. These features are statistical representations of sound parameters relevant to the exertion, tone, rhythm, and structure of the vocalizations.

Machine Learning-Based Classification: Supervised learning methods were then used to classify vocalizations into known categories, such as healthy versus unhealthy, behavioral types, and stress responses. The models combined traditional ensemble methods with neural and deep learning methods.

The pipeline converts raw acoustic input to measurable indices pertaining to poultry health and welfare. This permits real-time monitoring and/or automatic monitoring integration within broader sensor networks that include environmental and physiological data streams.

### 2.3. Feature Extraction and Classification Framework

In the next phases of the process, the audio signals obtained from the acquisition went through a processing phase to extract salient acoustic features for classification. The features focused on time-domain and frequency-domain characteristics that represent vocal effort, spectral energy distribution, and structure of the signal itself.

Some of the audio features extracted include the following:

Mel-Frequency Cepstral Coefficients or MFCCs [42] with their first and second derivatives. MFCC features were computed using 40 coefficients with a Hanning window, an FFT size (n_fft) of 2048 samples, a hop length of 512 samples (corresponding to 75% overlap), and a window length equal to the FFT size.

Spectral features such as contrast, centroid, bandwidth, and roll-off,

Temporal features such as zero-crossing rate (ZCR),

Statistical descriptors, including mean, standard deviation, skewness, and kurtosis for each feature vector.

All features were normalized by either min-max scaling or z-score standardization depending on the classification model. Supervised machine learning models were used to classify vocalizations according to health status, behavior, or stress conditions.

**Figure 2:**
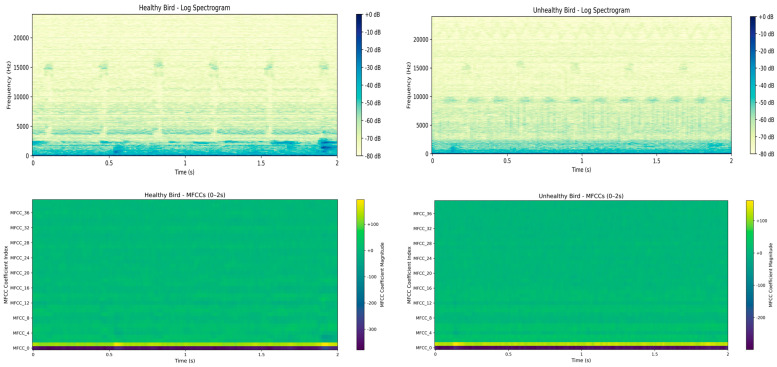
Log spectrograms and MFCC for healthy and unhealthy birds (0–2 s). Unhealthy vocalizations show reduced spectral richness and elevated low-frequency energy, indicating vocal strain.

These features extracted such as MFCCs, spectral contrast, and zero-crossing rate give a compact statistical representation of the frequency spectrum, energy distribution, and temporal dynamics of the vocal signals. For example, while MFCCs are beneficial in modeling the perception auditory scale and are sensitive to changes in vocal tracts, spectral contrasts capture the harmonic structure, and ZCR reflects the irregularity of the signal. Those properties have biological influences from factors like respiratory health, arousal, and stress. This translates raw audio into intelligible feature spaces through which machine learning models learn and relate distinct vocal behaviors to physiological and behavioral conditions in poultry. Figure 2 illustrates the spectral differences observed between healthy and unhealthy birds and supports our approach.

Models evaluated included Random Forest, CatBoost, HistGradientBoosting, MLPClassifier (Neural Network), AdaBoost, and TabNet (deep learning model optimized for tabular data).

### 2.4. Statistical Analysis

To assess feature relevance and class separability, independent two-sample *t*-tests were performed for each extracted feature at the significance level of *p* < 0.05. Feature importance was further assessed with model-specific techniques that include Gini importance (tree-based models), permutation importance, and attention weights (TabNet). 

For the analysis of inter-feature relationships as well as inter-metric relationships, Pearson correlation coefficients were calculated. Visualization tools such as heatmaps, violin plots, and time-series graphs were used to interpret the statistical and biological relevance of the data. 

### 2.5. Modeling Workflow and Cross-Validation Strategy

Following the completion of feature extraction and normalization, the acoustic data proceeded into the full pipeline of supervised classification. Multiple machine learning models were implemented to ensure diverse representational capabilities. These include tree-based ensemble methods such as Random Forest, Gradient Boosting, AdaBoost, Extra Trees, and HistGradientBoosting, as well as advanced models like CatBoost, Multi-Layer Perceptron (MLPClassifier), and TabNet. These classifiers were chosen on the basis of having been tested in high dimensionality, noisy, and nonlinear feature spaces, which is a norm in bio-acoustic data.

The machine learning models chosen in the research are deliberate mixtures of ensemble-based classifiers, neural networks, and hybrid deep learning architectures for tabular input. Such wide diversity matched the rather different kinds of extracted acoustic features that were mixtures across time- and frequency-domain descriptors and statistical moments. Random Forest and HistGradientBoosting were selected to apply their robustness to noisy inputs, capability to model non-linear interactions, and interpretability by importance feature metrics based on impurity. Such tree-based models are well-suited for heterogeneous datasets and have been widely used in the analysis of biological or medical signals. CatBoost is a powerful technique that uses gradient boosting specifically for its high performance over small to medium-sized structured datasets as it forms categorical or ordinal feature encoding (like call-type labels) handling efficiently and also reduces overfitting by ordered boosting and regularization ability. It also has built-in tools for evaluating feature contribution, thus increasing model transparency. The multi-layer perceptron (MLP) was specifically included for the modeling of deeper, non-linear dependencies among acoustic features that otherwise may not be well modeled by the tree-based approaches. It is particularly applicable in cases where such profiles are jointly related to physiological traits through the interaction of spectral and temporal descriptors. TabNet was evaluated as the deep learning model optimized specifically for tabular datasets. The salient mechanism of sequential attention makes the model learn a sparse and interpretable mask over input features at each decision step, providing an alternative to black box neural networks and classical tree ensembles. TabNet also opens up future scalability to larger, continuously streaming audio data-requiring architectures that can be realized in real-time edge-computing deployments. This model set was selected to balance accuracy, interpretability, and computation feasibility across the varied experimental conditions involved in this work.

In addition to static classification models, we included an LSTM network for the task of stress detection on Dataset 3. This dataset consisted of recordings across weeks and was therefore longitudinal. Unlike health or behavior classifications, this particular dataset captures temporal evolution over the vocal responses to stress. LSTMs are used since they are very suitable for retaining temporal dependencies and modeling sequential patterns of input data over the long run, settling these issues very well for time-series input data. To study the dynamic changes in vocal features over time such as habituation to stress or delayed responses, which are difficult for models lacking temporal memory to capture, LSTM would be most appropriate. Hence, aligned with both the nature of the biology and that of the experiment, we were able to identify stressor-specific trajectories within vocalization patterns using LSTM.

Every model was evaluated using stratified five-fold cross-validation to ensure a balanced presence of classes across the folds and to avoid any biases on learning arising due to uneven share of class distributions. During every fold, 80% of the data was used to train the models while the remaining 20% served validation. This procedure was carried out five times, and therefore, the final performance metrics were computed as an average across the folds. Thus, the whole exercise would provide robustness against variance in the training-test splits, and thus more reliable estimates of model generalization.

To give a really broad view of classification performance, a collection of standard supervised learning metrics was chosen that took into account the fact of bias and misleading prediction errors that occur in biological data. Accuracy indicates how many instances were correctly classified in overall proportion, but it may become misleading in contexts of imbalanced datasets where one class dominates others. Hence, we report precision (TP/predicted positives), recall (TP/actual positives), and F1-score (the harmonic mean of precision and recall) since they give weight to false positives and false negatives, depending on which carry more consequence in the immediate application when stressed or diseased birds could be misclassified more than possibly healthy ones. We further include Cohen’s Kappa, which measures agreement between predicted and true labels while accounting for chance, and Matthews Correlation Coefficient (MCC), which provides a balanced measure of binary classification performance even under skewed class distributions. Such metrics are highly recommended for biomedical and bio-acoustic classification tasks due to their resistance against imbalanced data and interpretability.

Hyperparameter tuning (Table 1) was carried out by RandomizedSearchCV, each model with different parameter grid specifications.

The optimal configuration for every model was chosen based on validation accuracy during three-fold cross-validation, followed by evaluation under five-fold stratified cross-validation. We explored grid search and Bayesian optimization with Optuna but maintained a randomized search for tuning hyperparameters. This made it easier to keep things consistent and compare the different classifiers fairly. All the input features were normalized using Z-score, except for TabNet since it can handle raw tabular data on its own. Gini importance was used to measure how important each feature was in the tree models. This helped us identify which acoustic features are linked to health and stress so that the approach makes sense biologically. Preliminary exploration was performed into advanced methods of interpretability such as TabNet attention weights and permutation-based importance; future developments will build upon such methods toward furthering agreement between model decisions and domain-appropriate physiological insights.

All modeling experiments were implemented using Python 3.11 and were run within Google Colab environments, which provided GPU support for neural network models. This workflow was supported mainly by the following libraries: Scikit-learn for traditional machine learning, PyTorch (v2.7.0) for deep learning, CatBoost for gradients boosting that support categorical features, and PyTabNet for TabNet. The modeling workflow thus allowed reproducibility, robustness, and interpretability across datasets and classification tasks.

## 3. Results and Discussion

### 3.1. Dataset 1: Health Classification Based on Vocalizations

The complexity of this issue is demonstrated by the use of different models in the study (Table 2). The models CatBoost and HistGradientBoosting, as shown by the results in the confusion matrices and all the accuracy plots, are appropriate for the task as they were able to perform well. Both excel at non-linear and feature interaction comprehension, which is crucial in biological datasets. These models have high accuracy and recall which suggests that the data patterns that distinguish healthy and unhealthy birds are captured by these models.

The fact that HistGradientBoosting provides perfect classification accuracy as also per the confusion matrix proves its better performance for both categorical and numerical feature sets as well as its ability to adjust the tree-based learning structures for better performance. Compared with others, TabNet did not seem to perform well and this was because it employs a deep learning model, as such models tend to work best with larger datasets. Because of the size of the dataset, TabNet was not able to generalize well, even though it was very good at learning features. Alternatively, this does not discount its usefulness when applied to more significant datasets or cases with varying feature representations. From the feature importance analysis it is easy to determine what sound subsets of MFCCs were important, and feature 7 received the highest importance of 8.8%. This is consistent with the biological knowledge that states how stress or illness changes certain aspects of vocalization.

We know MFCC features are representations of the spectral content of vocalization, and their change usually results from a variance in a bird’s respiratory and vocal apparatus. For instance, 40 MFCC coefficients were computed per frame with the Librosa library in the dataset we worked with. For each audio clip, MFCC_0 values were averaged across frames to yield a single value per clip. Later, these obtained clip-level MFCC_0 values were pooled by health status, and the mean computed for each group. The results showed that sick birds registered an average MFCC_0 of −319.2, while healthy birds had an average of −337.6. A two-sample *t*-test showed that the difference was statistically significant (*p* < 0.05), indicating that vocal impairment owing to sickness can affect the spectral envelope of the sounds produced. Our results are consistent with previous studies indicating that MFCCs are noninvasive measures of stress or disease in animal vocalizations [43].

**Figure 3:**
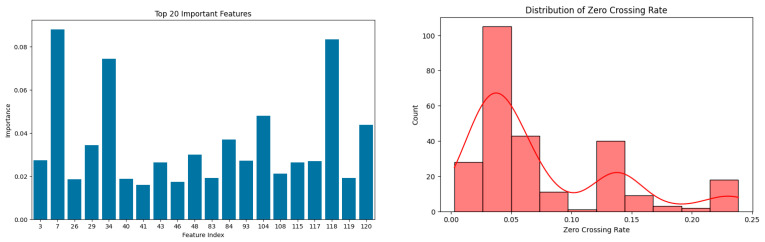
The top 20 most important features (**left**) ranked by their contribution to classification performance, with MFCC-based features showing the highest influence. Distribution of zero-crossing rate (**right**) across samples reveals increased variability and skewness in vocalizations.

The zero-crossing rate (ZCR) showed more dispersion among unhealthy birds indicating irregularity in the signals or instability in vocalization as seen in Figure 3. Using Gini importance averaged over five-fold cross-validation measures, these features were ranked and also confirmed through statistical comparisons that emphasize variation across health conditions. Most of the top 20 consisted of low-order MFCC mean and standard deviation measures (e.g., MFCC_2-MFCC_8), along with spectral contrast means and standard deviations (from bands 0 to 6), and signal-level descriptors, such as zero-crossing rate (ZCR). These cover mel-scale energy distribution, harmonic structure, and waveform irregularity, respectively, and ranked high across the folds. A different, but equally important feature, spectral contrast, was significantly lower in unhealthy birds (15.12 vs. 15.95, *p* < 0.05). This parameter measures the degree of sharpness and clarity of a sound’s frequency spectrum. The reduction in spectral contrast in unhealthy birds indicates some deterioration in the harmonic structure of their bird song.

Biologically, this may occur due to respiratory problems or other pathological conditions affecting the larynx, resulting in a greater degree of blur of distinctly recognizable harmonic tones. This can be accounted for by strained breathing or turbulent breathing patterns due to respiratory illness or any other medical condition. The responsiveness of ZCR captures the overall variability in sound production, which serves as a tell-tale in estimating the physiological demands and stress levels sustained by the birds.

### 3.2. Dataset 2: Behavioral Vocalization Classification 

Chicken vocalizations obtained from ChickenLanguageDataset were classified using five-fold cross-validation as well as by comparing several metrics for measuring and comparing different models for assessing the generalization and robustness of the models. The behavioral call types studied were “Do_you_have_food”, “eating”, “greeting”, “hungry”, “tidbitting_hen”, and “where_is_everyone”, and are associated with different physiological or social contexts. As seen in Table 3, overall classification accuracy for the model that performed best using HistGradientBoosting attained 80.4% and a robust macro F1-score of 0.807, which indicates an appropriate balance in sensitivity and precision across all the types of vocalizations.

**Figure 4:**
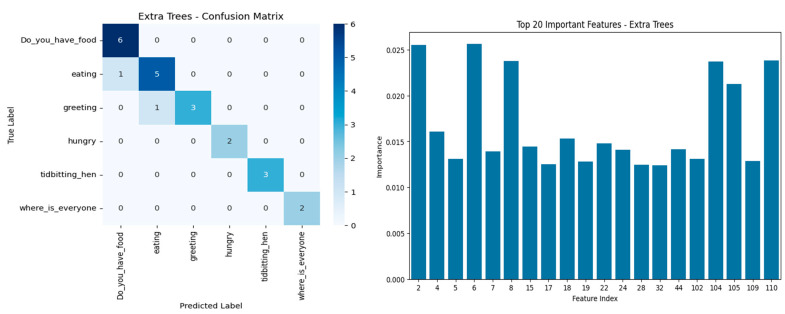
Confusion matrix (**right**) and top 20 feature importance plot (**left**) for Extra Trees. Clear classification is observed for distinct calls like “Do_you_have_food” and “tidbitting_hen.” Key features include MFCCs and spectral contrast indices, highlighting their role in vocal pattern discrimination.

Confusion matrices of the top models (Figure 4), Extra Trees and HistGradientBoosting, demonstrated a strong classification capability of “tidbitting_hen”, “where_is_everyone”, and “hungry” categories with little or no misclassifications. “Greeting” and “eating” are rated for moderate confusion with respect to adjacent behavioral labels in the context, showing that the two calls share a considerable amount of acoustic characteristics. Thus, Extra Trees had total classification in “Do_you_have_food” and “hungry” but could not perform well in “greeting”, whereas HistGradientBoosting showed stronger generalization across all categories.

Feature importance analysis revealed that several low-order MFCC coefficients (e.g., Features 2, 4, 6, 7, and 8, corresponding to MFCC_2 to MFCC_8 means) were among the most informative acoustic features. These coefficients usually capture mel-frequency spectral envelope variations between closely linked vocal resonance and energy patterns in animal vocalizations. This included the MFCCs, with the Spectral Contrast Bands 0, 1, and 6 (Features 104, 105, and 110) also listed among the top contributors. In fact, MFCC features showed clear dissimilarity between different vocalizations in our dataset, which is in line with their established role in detecting distress calls in poultry [43]. The dimensional timbral richness and harmonic structures allowed models to differentiate complex social calls from simple feeding cues. Correlation heat maps for MFCCs show moderate to low redundancy in most features, indicating efficacy in the spread of features without serious collinearity.

Several performance criteria beyond simple raw accuracy were used for the statistical evaluation across the models, including Cohen’s Kappa, Matthews Correlation Coefficient (MCC), and log loss difference. HistGradientBoosting and CatBoost scored the most on agreement (Cohen’s Kappa > 0.83).

This shows the reliability of the models against class imbalance. Furthermore, the very high MCC values of above 0.89 for these models confirmed their predictions would be very reliable, particularly in categories of low sample size. MLPClassifier initially showed promise but performed poorly after cross-validation (Cohen’s Kappa = 0.18, Log Loss Difference = −6.11), suggesting possible overfitting to training distributions during cross-validation or miscalibration on the minority classes. Overall, log loss differences between the models emphasized the robustness of HistGradientBoosting and Extra Trees against calibration.

**Figure 5:**
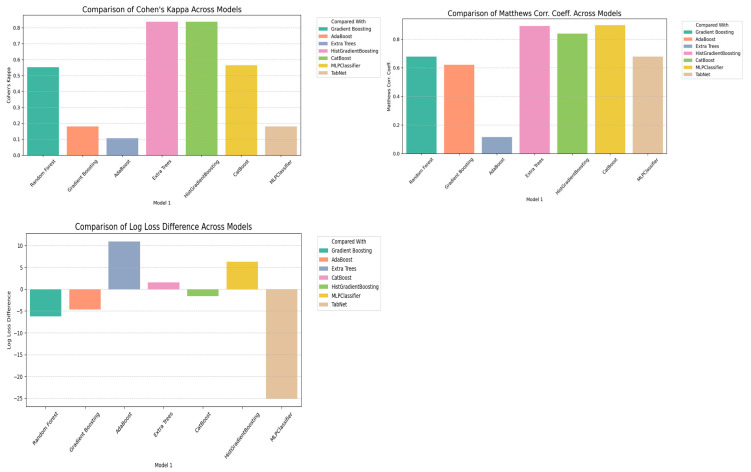
Statistical comparison of classifier performance across models using Cohen’s Kappa (**top-left**), Matthews Correlation Coefficient (**top-right**), and log loss difference (**bottom**). HistGradientBoosting and CatBoost exhibit highest agreement and predictive robustness across multiple metrics.

Cohen’s Kappa, MCC, and log loss were the statistical comparisons among models represented pictorially in Figure 5. The agreement among models and across statistical measures was further evaluated in a correlation heatmap. The moderate to strong positive correlation between MCC and Cohen’s Kappa (r = 0.75) suggested similar directions regarding reliability-based metrics. On the contrary, a negative correlation between MCC and log loss (r = −0.24) indicates that there is a trade-off between the penalties made for a misclassification instance and the confidence of that decision. Such findings thus provide multi-pronged interpretations of classifier robustness.

These MFCC feature correlation plots further proved that key features across samples maintained moderate independence, especially indices 2, 6, 8, 102, and 104, in accordance with biological relevance and the feature selection by tree-based models. Given the varying social and distress calls of chickens, the spectral and cepstral characteristics were important for effectively classifying between vocal behaviors. These results endorse the utility of high-end ensemble classifiers and MFCC-derived features for non-invasive, scalable behavioral monitoring in poultry.

### 3.3. Dataset 3: Health Classification Based on Vocalizations

One of the most highlighted observations was the separation trends between the two treatment groups: Trt1 and Trt2. This spreading indicates the inherent differences in the perception and effect of chicken species against different types of stressors. For example, Trt1 (opening of an umbrella) is a type of stressor that is visually induced. The response is normally characterized by surprise and triggers an acute response. Often these responses are defined by quick, sharp changes in vocalization patterns, which is indicated in the initial weeks of the study.

Contrarily, Trt2 (dog barking) is an auditory stressor that is more sustained, and in this instance, the vocalizations are reflective of a prolonged adaptation process. Auditory stressors engage different sensory and neurological pathways compared with visual stressors, consequently, yielding distinct responses. This differentiation illustrates that when analyzing vocalization data, the nature of the stressor has to be accounted for, as individual animals may be differentially responsive based on the type and timing of stressors. Variations in the traits of vocalizations over weeks also portray the shifting nature of stress responses. The sharp oscillations in many of these features during these early weeks suggest that the response is possibly driven by the novelty of the stressors and hence, the sensitivity of the birds during these first encounters. 

After some time, the trends stabilize away, suggesting a process of habituation. Habituation is a well-studied phenomenon in animal behavior: Each time an animal confronts the same stressor, its response diminishes. This has also been interpreted to indicate reduced stress levels on the part of the chickens since they have become accustomed. Alternatively, it may be interpreted as physiological responses adjusted to cope better with stress. Such adaptations are crucial for survival, more so in commercial poultry farming, where animals are often faced with different stress-inducing stimuli. That being said, while some features have settled down with time, others show a continued high standard of variability. Constant variability may indicate individual variability within birds, as not all animals respond to stressors in the same manner. Some of the most important factors that work to influence an individual’s response to an environmental challenge include genetic endowment, age, social rank, and past experiences. Such differences must be taken into account when devising any stress-reducing interventions, as any given intervention may not be equally effective for the entire herd of animals.

**Figure 6:**
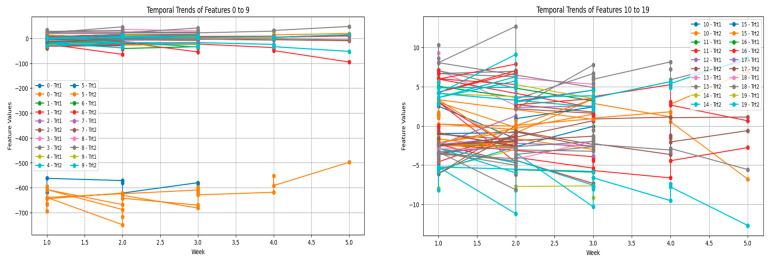
Temporal trends in acoustic features across weeks for Trt1 (umbrella opening) and Trt2 (dog barking). The (**left plot**) (features 0–9) shows early oscillations and subsequent stabilization, while the (**right plot**) (features 10–19) reveals persistent variability, indicating stressor-specific and individual response patterns.

The further grouping of features into segments for analyses offers further revelations concerning the duplicity of vocalization patterns. Thus, averaging the values within the feature groups reveals much broader trends that otherwise would have been shrouded in noise from the individual features. The segregation of Trt1 and Trt2 through these groups further strengthens that vocalization patterns reflect stressor-specific adaptations as seen in Figure 6. In addition, because of grouping, the analysis of high-dimensional data is spanned out better; thus, researchers may focus on the most wanton patterns.

These trends may reflect underlying physiological mechanisms of vocal control, particularly autonomic modulation of the syrinx. Vocalization is mediated through the syrinx, the avian voice organ, which is under autonomic control. During stressful periods, there are changes in the autonomic balance with corresponding changes in pitch, frequency, and amplitude. More than that, these changes are not random, because they reveal an animal’s internal state. For example, being higher-pitched or more frequent evokes a picture of heightened arousal or misery. The most distinct trends in Trt1 and Trt2 are likely to disturb the congruency of how these stressors affect the autonomic regulation of the syrinx.

**Figure 7:**
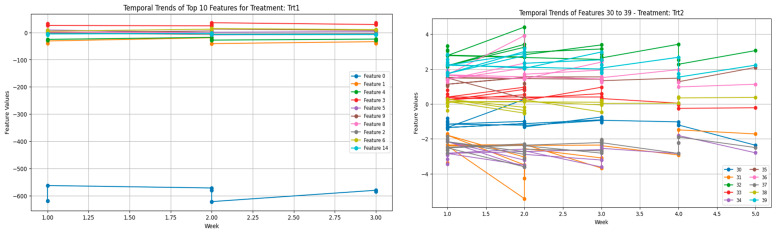
Temporal trends in selected acoustic features under individual treatments: Trt1 (**left**) and Trt2 (**right**). Feature dynamics in Trt1 remain stable or adapt rapidly, while Trt2 elicits higher variability and prolonged divergence across features, indicating differential stressor impact on vocal behavior.

Adding to this is the time-embedding in a dataset: by introducing times, more complications arise. Many factors, including the age and developing stage of the birds, might affect the week-to-week changes in vocalization features. In younger birds, vocalization is much more variable owing to their relatively undeveloped or immature stress handling and behavioral acclimatization. This is probably why the more volatile feature trends were observed during the earlier weeks of these plots (Figure 7). The more the young birds grow, the more control they have over their reactions, perhaps because their systems develop cognitive profiles to cope with stress well. The flock social dynamics that might enable that can be a variable, i.e., chickens are social, and their vocalizations function along that path of communication. 

**Figure 8:**
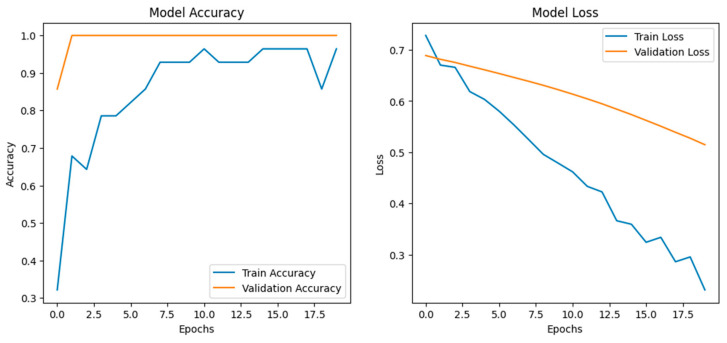
Training and validation performance of the LSTM model for stress classification in poultry. The (**left panel**) shows accuracy curves indicating rapid convergence and high generalization across epochs. The (**right panel**) displays steadily declining training and validation loss (binary cross-entropy), confirming model stability and effective learning dynamics without overfitting.

We employed an LSTM-based deep learning model to assess stress-induced vocalization patterns (Figure 8) under two experimental conditions: visual stress (e.g., umbrella opening) and auditory stress (e.g., dog barking). Extracted MFCC features from preprocessed audio clips were normalized and reshaped for time series modeling. The architecture consisted of two LSTM layers (128 and 64 units) with batch normalization and dropout layers for regularization, followed by dense layers using ReLU and sigmoid activations for binary classification. The training was carried out utilizing the Adam optimizer with binary cross-entropy loss, applying early stopping and learning rate scheduling strategies based on validation loss. During 20 epochs, the model converged rapidly, achieving high accuracies (greater than 95 percent) for both training and validation curves, and showed no signs of overfitting as validated by steadily decreasing loss values. Based on the biological implications, the performance of the model indicates clear acoustic variation between vocalizations depending on the types of stress applied. The high accuracy further suggests that chickens possess structured and distinguishable vocal responses that reflect the context of the stressor. Visual stress would result in an abrupt change in acoustic features typical of acute arousal, whereas auditory stress would result in prolonged variability, likely representative of a longer time horizon for adaptation. These patterns are likely under the control of the autonomic nervous system, with modulation reflected by their emotional or physiological state.

An altered vocalization pattern due to stress can potentially modify flock behavior; an example would be a dominant bird that would voice certain calls to maintain its status, while subordinate chickens might show more stressed vocalizations. Among other reasons, the welfare of the whole flock can indeed change from the chain of cause and effect manifesting from these dynamics, which action points toward a more holistic understanding of poultry stress and management. Such different temporal patterns have practical implications for poultry farming. Monitoring these vocalization patterns provides a noninvasive way to assess the welfare of chickens. Knowing the exact conditions that create vocal patterns of stress will allow farmers to ameliorate them. When, for instance, certain stressors consistently lead to an increase in vocalization variability, this can indicate that the birds are very distressed. Addressing such a stressor, whether through its removal, environmental change, or manipulation of the way chickens were handled by personnel, is vital for animal welfare and productivity. 

## 4. Limitations and Future Directions

Classification of poultry vocalizations based on acoustic characteristics remains constrained by several key limitations. Although the datasets used represent different behavioral and physiological conditions, their overall scale and ecological diversity are relatively limited. Background noise, overlapping calls within group-housed settings, and class imbalance continue to pose challenges for robust model training and generalization, even after preprocessing and stratified sampling efforts. The lack of concurrent physiological or biochemical ground-truth data restricts the depth of biological interpretation, particularly when linking acoustic features to welfare-relevant internal states.

Future directions include the integration of multimodal sensor data to enrich contextual understanding. Combining acoustic features with environmental parameters such as temperature, humidity, and gas concentrations, as well as visual inputs related to movement and posture, could enable a more comprehensive welfare assessment framework. Advancements in edge computing and TinyML offer pathways for real-time, on-farm deployment of acoustic models using low-power, cost-effective devices—minimizing latency and reliance on cloud infrastructure. Incorporating self-supervised and few-shot learning approaches would further improve model adaptability in data-scarce conditions, especially for underrepresented behaviors or farm environments. Extending the framework to additional poultry species or livestock systems, including ducks, turkeys, or quail, may also broaden the applicability and validate the generalizability of vocalization-based welfare monitoring. Collectively, these directions represent promising avenues for scaling acoustic sensing into practical, interpretable, and welfare-oriented solutions in precision livestock farming.

## 5. Conclusions

The present study demonstrates the feasibility and effectiveness of employing acoustic sensing, combined with statistical feature analysis and interpretable machine learning models, for non-invasive poultry welfare assessment. By examining vocalizations across health, behavioral, and stress-related contexts, the framework offers a comprehensive approach to detecting biologically relevant variations in sound signatures. The integration of mel-frequency cepstral coefficients, spectral features, and temporal dynamics enabled robust classification of welfare indicators, with ensemble methods and LSTM models achieving high predictive performance. Crucially, the interpretability of model outputs allowed for alignment with established physiological and behavioral knowledge, thereby enhancing both scientific validity and practical relevance. The behavioral classification revealed acoustically distinct call types, while stressor-specific temporal trends in vocal patterns underscored the capacity of acoustic signals to capture nuanced welfare states. Taken together, these findings support the deployment of acoustic monitoring as a scalable, real-time, and ethically aligned solution within precision livestock farming systems. The methodological rigor and biological grounding of this study offer a foundation for future work on deploying sensor-based tools that prioritize both data interpretability and animal well-being in commercial poultry operations. This approach holds practical implications for commercial poultry operations by enabling scalable, real-time monitoring of flock health and welfare using affordable acoustic sensors and interpretable machine learning models.

## Figures and Tables

**Table 1:** Hyperparameters Tuned for Each Classifier.

Classifier	Hyperparameters Tuned
Random Forest	n_estimators ∈ [100, 200], max_depth ∈ [None, 10, 20]
Extra Trees	n_estimators ∈ [100, 200], max_depth ∈ [None, 10, 20]
Gradient Boosting	learning_rate ∈ [0.01, 0.1], n_estimators ∈ [100, 200], max_depth ∈ [3, 5, 7]
AdaBoost	learning_rate ∈ [0.01, 0.1], n_estimators ∈ [100, 200]
CatBoost	iterations ∈ [100, 200], learning_rate ∈ [0.01, 0.1], depth ∈ [4, 6, 8]
HistGradientBoosting	learning_rate ∈ [0.01, 0.1], max_depth ∈ [3, 5, 7], l2_regularization ∈ [0.1, 1]
MLPClassifier	hidden_layer_sizes ∈ [(50, 50), (100, 50)], activation ∈ [‘relu’, ‘tanh’], alpha ∈ [0.0001, 0.001]
TabNet	n_d, n_a ∈ [8, 16, 24], n_steps ∈ [3, 5, 7]

**Table 2:** Performance metrics (accuracy, precision, recall, F1-score) of baseline models for Dataset − 1.

Model	Accuracy	Precision	Recall	F1 Score
Random Forest	0.977	0.978	0.977	0.977
Gradient Boosting	0.973	0.974	0.973	0.973
AdaBoost	0.988	0.989	0.988	0.988
Extra Trees	0.988	0.989	0.988	0.988
HistGradientBoosting	0.992	0.993	0.992	0.992
CatBoost	0.985	0.985	0.985	0.985
MLPClassifier	0.988	0.989	0.988	0.988
TabNet	0.612	0.616	0.612	0.595

**Table 3:** Performance metrics (accuracy, precision, recall, F1-score) of baseline models for Dataset 2.

Model	Accuracy	Precision	Recall	F1 Score
Random Forest	0.804	0.803	0.804	0.802
Gradient Boosting	0.607	0.591	0.607	0.591
AdaBoost	0.464	0.533	0.464	0.457
Extra Trees	0.768	0.770	0.768	0.766
HistGradientBoosting	0.804	0.817	0.804	0.807
CatBoost	0.196	0.863	0.857	0.858
MLPClassifier	0.741	0.756	0.741	0.745
TabNet	0.214	0.291	0.214	0.193

## Data Availability

The datasets used in this study are publicly available: Chicken Vocalization for Health Detection: Available on Zenodo at https://zenodo.org/records/10433023; Stress Detection in Poultry: Available on Mendeley Data at https://data.mendeley.com/datasets/zp4nf2dxbh/1; Chicken Language Dataset: Available on GitHub at https://github.com/zebular13/ChickenLanguageDataset; All datasets are free to access and use under their respective open licenses.

## References

[1] Ungar E.D., Nevo Y. (2025). Rhythms, Patterns and Styles in the Jaw Movement Activity of Beef Cattle on Rangeland as Revealed by Acoustic Monitoring. Sensors.

[2] Gao G., Ma Y., Wang J., Li Z., Wang Y., Bai H. (2025). CFR-YOLO: A Novel Cow Face Detection Network Based on YOLOv7 Improvement. Sensors.

[3] Dewmini H., Meedeniya D., Perera C. (2025). Elephant Sound Classification Using Deep Learning Optimization. Sensors.

[4] Karaaslan M., Turkoglu B., Kaya E., Asuroglu T. (2024). Voice Analysis in Dogs with Deep Learning: Development of a Fully Automatic Voice Analysis System for Bioacoustics Studies. Sensors.

[5] de Carvalho Soster P., Grzywalski T., Hou Y., Thomas P., Dedeurwaerder A., De Gussem M., Tuyttens F., Devos P., Botteldooren D., Antonissen G. (2025). Automated detection of broiler vocalizations a machine learning approach for broiler chicken vocalization monitoring. Poult. Sci..

[6] Adebayo S., Aworinde H.O., Akinwunmi A.O., Alabi O.M., Ayandiji A., Sakpere A.B., Adeyemo A., Oyebamiji A.K., Olaide O. (2023). Enhancing poultry health management through machine learning-based analysis of vocalization signals dataset. Data Brief.

[7] Zhao S., Cui W., Yin G., Wei H., Li J., Bao J. (2023). Effects of different auditory environments on behavior, learning ability, and fearfulness in 4-week-old laying hen chicks. Animals.

[8] Srinivasagan R., El Sayed M.S., Al-Rasheed M.I., Alzahrani A.S. (2025). Edge intelligence for poultry welfare: Utilizing tiny machine learning neural network processors for vocalization analysis. PLoS ONE.

[9] Cuan K., Zhang T., Li Z., Huang J., Ding Y., Fang C. (2022). Automatic Newcastle disease detection using sound technology and deep learning method. Comput. Electron. Agric..

[10] Cuan K., Zhang T., Huang J., Fang C., Guan Y. (2020). Detection of avian influenza-infected chickens based on a chicken sound convolutional neural network. Comput. Electron. Agric..

[11] Du X., Carpentier L., Teng G., Liu M., Wang C., Norton T. (2020). Assessment of laying hens’ thermal comfort using sound technology. Sensors.

[12] Hassan E., Elbedwehy S., Shams M.Y., Abd El-Hafeez T., El-Rashidy N. (2024). Optimizing poultry audio signal classification with deep learning and burn layer fusion. J. Big Data.

[13] Ginovart-Panisello G.J., Iriondo I., Panisello Monjo T., Riva S., Cancer J.C., Alsina-Pagès R.M. (2024). Acoustic detection of vaccine reactions in hens for assessing anti-inflammatory product efficacy. Appl. Sci..

[14] Ginovart-Panisello G.J., Iriondo Sanz I., Panisello Monjo T., Riva S., Garriga Dicuzzo T., Abancens Escuer E., Alsina-Pagès R.M. (2022). Trend and representativeness of acoustic features of broiler chicken vocalisations related to CO_2_. Appl. Sci..

[15] Ginovart-Panisello G.-J., Alsina-Pagès R.M., Panisello Monjo T. (2020). Acoustic description of bird broiler vocalisations in a real-life intensive farm and its impact on animal welfare: A comparative analysis of recordings. Eng. Proc..

[16] Ginovart-Panisello G.-J., Alsina-Pagès R.M., Iriondo Sanz I., Panisello Monjo T., Call Prat M. (2020). Acoustic description of the soundscape of a real-life intensive farm and its impact on animal welfare: A preliminary analysis of farm sounds and bird vocalisations. Sensors.

[17] Ginovart-Panisello G.J., Iriondo I., Panisello Monjo T., Riva S., Garcia R., Valls J., Alsina-Pagès R.M. (2024). Acoustic detection of the effects of prolonged fasting on newly hatched broiler chickens. Comput. Electron. Agric..

[18] Li Z., Zhang T., Cuan K., Fang C., Zhao H., Guan C., Yang Q., Qu H. (2022). Sex detection of chicks based on audio technology and deep learning methods. Animals.

[19] Schober J.M., Merritt J., Ulrey M., Yap T.Y., Lucas J.R., Fraley G.S. (2024). Vocalizations of the Pekin duck (Anas platyrhynchos domesticus): How stimuli, sex, and social groups affect their vocal repertoire. Poult. Sci..

[20] Collins S.A., Herborn K., Sufka K.J., Asher L., Brilot B. (2024). Do I sound anxious? Emotional arousal is linked to changes in vocalisations in domestic chicks (*Gallus gallus* dom.). Appl. Anim. Behav. Sci..

[21] Jung D.-H., Kim N.Y., Moon S.H., Jhin C., Kim H.-J., Yang J.-S., Kim H.S., Lee T.S., Lee J.Y., Park S.H. (2021). Deep learning-based cattle vocal classification model and real-time livestock monitoring system with noise filtering. Animals.

[22] Wang K., Wu P., Cui H., Xuan C., Su H. (2021). Identification and classification for sheep foraging behavior based on acoustic signal and deep learning. Comput. Electron. Agric..

[23] Ivanenko A., Watkins P., van Gerven M.A., Hammerschmidt K., Englitz B. (2020). Classifying sex and strain from mouse ultrasonic vocalizations using deep learning. PLoS Comput. Biol..

[24] Premoli M., Baggi D., Bianchetti M., Gnutti A., Bondaschi M., Mastinu A., Migliorati P., Signoroni A., Leonardi R., Memo M. (2021). Automatic classification of mice vocalizations using ML and CNNs. PLoS ONE.

[25] Bianco M.J., Gerstoft P., Traer J., Ozanich E., Roch M.A., Gannot S., Deledalle C.A. (2019). Machine learning in acoustics: Theory and applications. J. Acoust. Soc. Am..

[26] Gavojdian D., Cziszter L.T., Popovici D., Csosz A., Ivan M., Halaszi R., Biro D. (2024). BovineTalk: A deep learning and explainable AI-based approach for dairy cattle vocal classification under isolation stress. Animals.

[27] Sattar F. (2022). A context-aware method-based cattle vocal classification system using multi-resolution cochleagram features. Comput. Electron. Agric..

[28] Schneider S., Hammerschmidt K., Dierkes P.W. (2022). Introducing the Software CASE (Cluster and Analyze Sound Events) by Comparing Different Clustering Methods and Audio Transformation Techniques Using Animal Vocalizations. Animals.

[29] Lavner Y., Pérez-Granados C. (2024). Editorial: Computational bioacoustics and automated recognition of bird vocalizations. Front. Bird Sci..

[30] Mutanu L., Gohil J., Gupta K., Wagio P., Kotonya G. (2022). A Review of Automated Bioacoustics and General Acoustics Classification Research. Sensors.

[31] Terasaka D.T., Martins L.E., dos Santos V.A., Ventura T.M., de Oliveira A.G., Pedroso G.S.G. (2024). Audio segmentation to build bird training datasets. Inst. De Comput. UFMT.

[32] Sethi S.S., Bick A., Chen M.-Y., Crouzeilles R., Hillier B.V., Lawson J., Lee C.-Y., Liu S.-H., de Freitas Parruco C.H., Rosten C.M. (2024). Large-scale avian vocalization detection delivers reliable global biodiversity insights. Proc. Natl. Acad. Sci. USA.

[33] McGinn K., Kahl S., Peery M.Z., Klinck H., Wood C.M. (2023). Feature Embeddings from the BirdNET Algorithm Provide Insights into Avian Ecology. Ecol. Inform..

[34] Ghani B., Kalkman V.J., Planqué B., Vellinga W.-P., Gill L., Stowell D. (2024). Generalization in Birdsong Classification: Impact of Transfer Learning Methods and Dataset Characteristics. arXiv.

[35] Mørk J., Bovbjerg H.S., Kiss G., Tan Z.-H. (2024). Noise-robust keyword spotting through self-supervised pretraining. arXiv.

[36] Sasek J., Allison B., Contina A., Knobles D., Wilson P., Keitt T. (2024). Semiautomated generation of species-specific training data from large, unlabeled acoustic datasets for deep supervised birdsong isolation. PeerJ.

[37] Brydinskyi V., Sabodashko D., Khoma Y., Podpora M., Konovalov A., Khoma V. (2024). Enhancing automatic speech recognition with personalized models: Improving accuracy through individualized fine-tuning. IEEE Access.

[38] Shirahata Y., Park B., Yamamoto R., Tachibana K. (2024). Audio-conditioned phonemic and prosodic annotation for building text-to-speech models from unlabeled speech data. arXiv.

[39] Tosato G., Shehata A., Janssen J., Kamp K., Jati P., Stowell D. (2023). Auto Deep Learning for Bioacoustic Signals. arXiv.

[40] Collias N.E., Joos M. (1953). The spectrographic analysis of sound signals of the domestic fowl. Behaviour.

[41] Michaud F., Sueur J., Le Cesne M., Haupert S. (2023). Unsupervised classification to improve the quality of a bird song recording dataset. Ecol. Inform..

[42] Prabakaran D., Sriuppili S. (2021). Speech processing: MFCC based feature extraction techniques—An investigation. J. Phys. Conf. Ser..

[43] Tao W., Wang G., Sun Z., Xiao S., Wu Q., Zhang M. (2022). Recognition Method for Broiler Sound Signals Based on Multi-Domain Sound Features and Classification Model. Sensors.

